# Do Popular Diets Impact Fertility?

**DOI:** 10.3390/nu16111726

**Published:** 2024-05-31

**Authors:** Maria Salvaleda-Mateu, Cristina Rodríguez-Varela, Elena Labarta

**Affiliations:** 1 IVI Foundation, Instituto de Investigación Sanitaria La Fe, 46026 Valencia, Spain; 2 Human Reproduction Department, IVI RMA Valencia, 46015 Valencia, Spain; cristina.rodriguez@ivirma.com (C.R.-V.); elena.labarta@ivirma.com (E.L.)

**Keywords:** diet, fertility, infertility, nutrition, lifestyle, conception, food

## Abstract

Infertility affects 15% of the population in developed countries, and its prevalence is increasing. Fertility can be influenced by different factors. Although key factors like maternal age cannot be changed, there is growing evidence that other modifiable factors, such as diet, can have an impact on fertility. Diet has become increasingly important in recent years for a number of reasons: the new trend toward a healthy lifestyle, the higher prevalence of certain digestive disorders, a lack of time that leads people to consume more prepared and processed food, and personal choice to not eat meat, among others. To meet these needs, several diets have recently become popular, such as the Mediterranean diet, known as the gold standard of health; the DASH diet, known for preventing hypertension; the Western diet, characterized by processed food; the ketogenic diet, characterized by low carbohydrate intake; and the vegetarian diet, which is the choice for people who do not eat meat or animal by-products. Diets present a unique composition characterized by the presence or absence of specific nutrients, which have also been associated with male and female fertility individually. This review assesses the impact of these diets and of macro- and micronutrients on both female and male fertility.

## 1. Introduction

Lifestyle and diet play crucial roles in influencing fertility by impacting various physiological and hormonal factors. A well-balanced diet rich in essential nutrients, vitamins, and minerals is fundamental for both women’s and men’s reproductive health [1]. Adopting a healthy lifestyle, including a nutritious diet, regular exercise, and stress management, can significantly enhance fertility outcomes for individuals or couples seeking to conceive [2].

Diet is a particularly key aspect of contemporary society because it plays a significant role not only in providing essential nutrients but also in social, cultural, and economic domains [3]. Diet is a modifiable factor, and various circumstances can influence eating behavior. For instance, income, the level of education, and even one’s partner’s dietary habits can influence an individual’s diet [4,5]. The choices made in terms of dietary preferences and habits are often shaped by these external factors. In a broader context, the globalization impact has been evidenced in recent decades because higher-income countries have shifted from their traditional dietary practices to adopt the Western diet, characterized by increased consumption of processed foods, high sugar intake, and the incorporation of trans fatty acids (TFAs) [6,7]. The popularity of this eating habit may be attributed to its availability in supermarkets and food chains [6] and also to its extensive marketing [8]. Nevertheless, this nutritional plan has frequently been deemed detrimental and linked with numerous illnesses and weight gain [9].

In fact, noncommunicable diseases (NCDs), also known as lifestyle diseases, have the highest global morbidity and mortality rate. NCDs are the result of a combination of genetic, physiological, environmental, and behavioral factors [10].

Promoting preventive measures by focusing on lifestyle modifications, especially diet, are gaining attention as a social trend [10,11,12].

Several diets have become popular in the past few years, with different patterns according to the objective: for example, healthy, anti-inflammatory, and weight loss diets, among others, such as the Mediterranean diet, the DASH diet, the vegetarian diet, the vegan diet, and the ketogenic diet [13].

**Figure 1:**
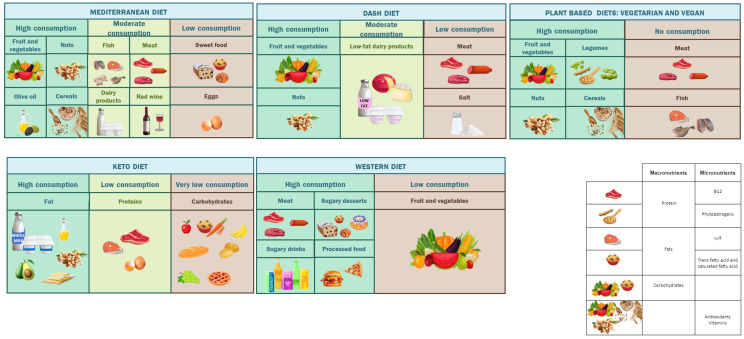
Composition of the Mediterranean diet, the DASH diet, the keto diet, plant-based diets (vegetarian and vegan), and the Western diet.

Furthermore, these diets are distinguished by their specific macronutrient and micronutrient compositions, which are illustrated in Figure 1, and have been linked individually with male and female reproductive function. It is noteworthy that other nutrients have also attracted the population’s attention; for instance, the gluten-free diet has gained considerable popularity due mainly to digestive disorders and is the third-most-searched diet on Google [13].

This narrative review summarizes all the information available to date on the effects of these trendy dietary patterns and the impact of macronutrients and micronutrients on female and male fertility.

## 2. Methodology

An in-depth review was conducted of all the scientific articles found in the bibliographic database PubMed and in Google Scholar using keywords such as “nutrition”, “fertility”, “ovarian function”, “endometrial function”, “semen parameters”, and “pregnancy”, among others. All the scientific works found suitable for this topic and research were included in this review.

## 3. Diets

Figure 1 in Section 1 summarizes the composition of the diets described next (the Mediterranean diet, the DASH diet, plant-based diets, the keto diet, and the Western diet).

### 3.1. Mediterranean Diet

The Mediterranean diet (MD) was first described in 1986 [14]. Since then, different variations of this nutrition plan have been developed. Nevertheless, the essential elements are consistent in them all. This diet consists mainly of high consumption of olive oil, vegetables, fruit, nuts, legumes, and cereals; moderate consumption of fish, meat, dairy products, and red wine; and low consumption of sweets and eggs [15].

This diet is considered the gold standard for health. It has been linked with several health benefits, particularly the improvement of metabolic diseases, such as diabetes and insulin resistance [16], which are, in turn, closely associated with infertility [17,18].

Regarding fertility, in a case–control study [19], 8619 women of reproductive age (20–45 years old) were followed and contacted for nutritional habits using food-frequency questionnaires (FFQs) and fertility-related data every 2 years over a 7-year period. Of this cohort, 485 women with proper collected data reported having visited a physician due to difficulties conceiving, and 1669 were selected as properly matched controls. After classifying the participants into four quartiles based on their MD adherence, greater adherence to this diet was associated with a lower probability of experiencing difficulty getting pregnant.

In terms of intermediate fertility outcomes in assisted reproductive techniques, there is a general consensus in observational studies using FFQs that maternal [20,21] and couples’ [22,23] MD adherence does not affect blastocyst quality. Kermack et al. [23] conducted a clinical randomized trial with 111 couples. The control group (56 couples) received sunflower seed oil for cooking, a spreadable paste based on sunflower oil, and a daily drink without omega 3 (ω3) and vitamin D. The intervention group (55 couples) received supplements to mimic the MD (olive oil for cooking and a daily drink with ω3 fatty acid and vitamin D) for 6 weeks. Although no differences in blastocyst quality were observed between both groups, improvements in embryo quality in the cleavage stage were reported. There is some controversy in the literature about the relation between MD adherence and other intermediate outcomes, such as fertilization rates and embryo yield. Previous works have found no association [20], but more recent studies have observed a slight benefit of maternal MD adherence for these parameters [24].

Regarding the key fertility parameters in assisted reproductive techniques, several observational studies have found a positive link between maternal MD adherence and pregnancy [20] and live birth rates [20,25]. Nevertheless, other works have failed to establish a relation between adherence to this diet and these key outcomes [21,24,26]. On couples’ MD adherence, Vujkovic et al. [22] have reported that high MD adherence improves the probability of pregnancy (OR 1.4, 95%CI 1.0–1.9).

In order to address these conflicting results, two recent meta-analyses were conducted to examine the impact of MD adherence on pregnancy and live births [27,28]. The meta-analysis conducted by Winter et al. [27], which included five studies in populations receiving assisted reproductive treatment, concluded that high MD adherence may increase clinical pregnancy and the live birth rate by approximately one-third (OR 1.27, 95%CI 0.82–1.98, *I*^2^ = 60%), which increased to two-thirds (OR 1.91, 95%CI 1.14–3.19, *I*^2^ = 43%) after excluding three studies considered to present a high risk of bias. Contrary to these results, Muffone et al. [28] could not establish a link between MD adherence and pregnancy outcomes (live births and clinical pregnancy), due to the heterogeneity of the studies. In the meta-analysis, only two studies that investigated the relation of MD adherence and the live birth rate were included, and a negative association was observed (OR 0.656, 95%CI 0.231–1.63, *I*^2^ = 83.16%). A slightly positive association between following this diet and clinical pregnancy based on five studies (OR 1.192, 95%CI 0.329–4.325, *I*^2^ = 93.8%) was reported. Nevertheless, as mentioned before, these results were invalidated because the meta-analysis showed high heterogeneity.

Regarding sperm parameters, the MD seems to be positive for sperm quality. In fact, an improvement in sperm parameters, mainly in motility, was seen in 137 men who followed an MD plan for 16 weeks [29]. This was further supported by the results of a recent meta-analysis, which showed a positive effect of MD adherence on sperm concentration, sperm progression, and sperm count [28].

### 3.2. Dash Diet

The Dietary Approaches to Stop Hypertension (DASH) diet is high in fruit, vegetables, and nuts and low in meat but is characterized by low salt levels and moderate consumption of low-fat dairy products [30]. This dietary plan has proved efficient in preventing and treating hypertension [31].

There is currently no direct association between the DASH diet and women’s fertility in terms of clinical pregnancy or live births [26]. Nevertheless, this nutritional plan has been indirectly linked with an improvement in ovarian morphology, as indicated by a slight reduction in the follicle count and ovarian volume [32]. Moreover, several studies have reported an indirect beneficial effect on some metabolic parameters that may be related to reproductive outcomes in women with polycystic ovary syndrome (PCOS), such as body mass index (BMI) reduction and insulin resistance enhancement [33,34].

The literature suggests that a general consensus exists about the positive correlation between DASH diet adherence and semen quality improvement [35,36,37]. According to several observational studies using FFQs, men who adhere more to this dietary plan have a significantly higher sperm concentration [35,36,37] and sperm count [37] compared to the men with lower adherence. However, there is still controversy about this topic because Salas-Huertos et al. [38] conducted an observational study that also used an FFQ and found no significant differences in the semen qualities between men with the greatest adherence to this diet plan and those with the lowest. To the best of our knowledge, this is the only study that has evaluated pregnancy and live birth rates in relation to DASH diet adherence in men, and no association was found.

### 3.3. Plant-Based Diets: Vegetarian and Vegan Diets

Plant-based diets consist of eating plans, with a high intake of vegetables, fruit, grains, and beans. These diets include vegan and vegetarian diets. The key factor in both these diets is no meat and fish intake. These types of nutritional habits are becoming more popular for ethical, health, and religious reasons. Although socially considered healthy, several nutritional deficiencies have been identified in vegan and vegetarian diets, including iron, zinc, vitamin D, and vitamin B12 deficiencies [39,40].

Data on female fertility and these diets are scarce and unclear. The results in the literature suggest that these eating plans have a negative effect on the menstrual cycle. However, it has not been possible to determine whether menstrual cycle changes are due to a vegetarian diet itself or due to nutritional deficiencies [41,42,43]. Conversely, a small prospective study compared subclinical ovulatory abnormalities in 23 vegetarian and vegan women to those in 22 omnivore women. It was observed that the vegetarian and vegan groups presented fewer subclinical ovulatory alterations than the omnivore group [44].

It is also worth noting that vegan and vegetarian diets are generally perceived as healthy but could also be unhealthy depending on the diet’s composition and the consumption of certain foods, such as refined grains and highly processed food. This could potentially affect fecundability. Lim et al. [45] observed in a cohort of 805 Asian women, who reported their dietary data on FFQs, that those with a higher adherence to a healthy plant-based diet (higher vegetable and fruit intake) took less time to get pregnant than the women who followed an unhealthy plant-based diet (high intake of fast food and sugary drinks). Finally, a recent meta-analysis examined sex hormone levels and concluded that it was not possible to associate the vegetarian diet with a positive or negative effect on fertility and reported only a reduction in estrone levels in the vegetarian population [46].

As far as pregnancy safety is concerned, women who follow a vegetarian diet during the gestational period have been documented as being at higher risk of having babies with a low birth weight. This has been linked with the fact that vegetarian women tend to gain less weight during pregnancy. However, no other adverse neonatal outcomes have been associated with following this type of diet during gestation [47,48].

There are conflicting data about how semen parameters may be affected by these types of nutritional plans. Some studies have observed better sperm quality in vegan men compared to nonvegan men [49], and others have indicated lower quality [50]. Moreover, a recent meta-analysis reported no conclusive effect of a vegetarian diet on semen characteristics [46].

On another note, in vegan and vegetarian men, different epigenetics and insufficient sperm hyperactivity have been reported [50,51]. The latter may indicate an alteration in calcium membrane channels [50]. Furthermore, changes in the sperm membrane, such as lower ω3 fatty acid levels, particularly docosahexaenoic acid (DHA), have been detected in young vegan men [52].

### 3.4. Keto Diet

The classic keto diet consists of 80% fat, 15% protein, and 5% carbohydrates. As fat is the main source of energy instead of carbohydrates, it allows the body to enter a state of ketosis, which is the main feature of this nutrition plan [53]. In this stage, ketone levels increase because, when glucose cannot be obtained from carbohydrates, the body uses fat as fuel for energy production, and the liver obtains the components required to produce ketones from this process. This stage has been observed during starvation periods or extremely low-carb diets [54]. The huge popularity of this nutritional plan might be because it has been shown to improve type 2 diabetes, has anti-inflammatory properties, and is associated with weight loss [55,56].

Research into the keto diet and fertility has focused mainly on obese and overweight women with PCOS. There is a general consensus that this dietary plan improves fertility in women with these conditions. Recently, Pandurevic et al. [57] conducted a randomized controlled trial that included 27 obese women with PCOS. The experimental group (*n* = 13) was on a ketogenic diet for 8 weeks, followed by 8 more weeks on a low-calorie diet. The control group (*n* = 14) followed a low-calorie Mediterranean diet for 16 weeks. A significant increase in spontaneous ovulation cycles, from 38.5% to 84.6%, was noted in the experimental group (*p* = 0.031), with an almost 50% increase after 16 weeks. The control group presented a 20% increase in spontaneous ovulation, which was not statistically significant. In line with these results, an improvement in menstrual regulation in 70% of the cases following the keto diet for 120 days has been recently reported [58]. Key fertility parameters, such as pregnancy and live births, also appear to improve in patients with these characteristics [59,60]. In the most recently published study, 12 obese women with PCOS followed a ketogenic diet for 14 ± 11 weeks after an unsuccessful in vitro fertilization (IVF) cycle. The authors observed that the ovarian outcomes (antral follicular count, number of oocytes, MII retrieved) of the nutritional intervention cycle were similar to those of the previous cycle. Yet, whereas no women had achieved pregnancy in the previous cycle, 8 out of 12 women became pregnant in the cycle following the keto diet, with seven live births [60].

A well-established improvement in sexual hormones, insulin resistance, and weight loss in obese women with PCOS following this diet has been reported in several publications, which might be the reason why fertility in these women is enhanced [61,62,63]. Moreover, mitochondrial function also appears to be enhanced in this type of diet, which is closely linked with reproduction function [64,65].

It is worth mentioning that this nutritional plan could be harmful for the body in the long term because it may also lead to deficiencies in certain important components and nutrients, such as fiber and vitamins [55]. Furthermore, following the keto diet during pregnancy in the animal model has proven to have a detrimental effect on the offspring’s neurological development and fertility [66,67,68].

### 3.5. Western Diet

The Western diet is characterized by its high intake of red and processed meats, unhealthy snacks, sweet foods, sugary desserts and drinks, and ultraprocessed foods (usually considered junk and fast food), along with low fruit and vegetable intake [69,70,71]. This diet is becoming increasingly prevalent in high-income countries and has been linked with numerous diseases [70,72].

This nutritional plan appears to be unrelated to female reproductive outcomes, according to the literature [19,27]. Nevertheless, different observational studies have shown a positive association between the highest adherence to the Western diet and a higher risk of PCOS and clinical features linked with this condition [73,74]. In fact, the growing popularity of this nutritional trend worldwide has been linked with a rise in both obesity and the prevalence of PCOS [75,76]. Medical practitioners have particularly observed an increment in this condition in middle-class women. While factors like a lack of exercise are suggested as potential contributors, the most supported reason is diet [77]. In animals, mostly nonhuman primates, several studies have reported a negative impact of this dietary pattern on fertility-related parameters. These include lower progesterone levels [78,79], fewer available blastocysts [80,81], and lower pregnancy rates [82] in females on this diet compared to those on healthier diets. Moreover, endometrial function has been reported to be impaired by the main components of this nutritional habit in rats and in vitro experiments [83].

The Western diet has frequently been associated with a lower semen concentration [84,85,86,87]. Arab et al. [88] conducted a meta-analysis that involved six studies. They concluded that the sperm concentration was significantly lower in participants with the highest adherence to the Western diet than in participants who adhered to a healthy nutritional plan (mean difference: 0.66, 95%CI 0.305–1.016, *p* < 0.001). Nevertheless, the association between the Western diet and the other semen parameters found in observational studies that have reported the dietary pattern using FFQs is unclear [84,85,86,89], and no correlation between semen quality and this diet appears in a few studies [69]. However, it is noteworthy that adherence to this dietary plan and some key components of the Western diet (sweet foods and processed meat) have been associated with more than double the risk of asthenozoospermia [90,91].

In the literature, several suggestions have been made as to how the Western diet may negatively affect fertility. First, it has been associated with weight gain and a higher BMI [92,93]. Albeit controversial, obesity and overweight have been linked with an impairment in women’s and men’s ability to conceive [94]. Moreover, this nutritional pattern is high in fats, especially TFAs, and also in carbohydrates, which may be involved in mechanisms that hamper reproductive outcomes [95,96]. This dietary pattern is also considered to be an inflammatory diet. The lower fiber and vitamin intakes reduce their anti-inflammatory effects on the body [97]. In fact, an alteration in intrafollicular fluid molecules in subjects who adhered to the Western diet has been reported, which suggests an inflammatory environment that could disrupt oocyte development [80].

## 4. Macronutrients

### 4.1. Fat

The relation between fertility and fat intake has been investigated, and a general consensus has been reached that the quality of fats is important for fertility. In female fertility, high-fat diets with nonspecified fat intake have been associated with ovulation disorders and obesity [98].

Regarding specific fats, TFAs have a negative effect [99,100,101]. It has been observed that when 2% energy is derived from TFAs instead of from carbohydrates, omega-6 fatty acids, and monounsaturated fats, the ovulatory infertility risk increases (RR 1.73, 95%CI 1.09–2.73; RR 1.79, 95%CI 1.11–2.89; and RR 2.31, 95%CI 1.09–4.87, respectively) [99]. With IVF outcomes, TFAs seem to lower both the fertilization rate and the number of available embryos but are not associated with clinical pregnancy [101]. It has also been suggested that TFAs may slightly increase fetal loss (OR 1.106, 95%CI 1.026–1.192) by down-regulating the PPAR-gamma gene, which is crucial for placental development [102], and may have a negative effect on fertility in individuals with insulin disorders through lipo-toxicity and insulin sensitivity [96,103].

A recent systematic review about ω3 and female fertility has observed a positive association between embryo and oocyte quality and women’s ω3 intake, although high levels of specific ω3 fats, eicosapentaenoic acid (EPA), and DHA appeared to lower the number of retrieved oocytes [104]. On the relation of key fertility parameters (pregnancy and live birth rates) and women’s ω3 consumption, several studies have been unable to establish a connection according to blood ω3 levels in natural pregnancy [105]. However, works with a study population who were IVF patients have found a positive correlation in both serum ω3 levels [106] and ω3 intake [107]. The most recent observational study has noted a positive, albeit nonsignificant, trend in live birth rates with higher ω3 intake and a significant increase in the live birth rate with the highest EPA and DHA intakes compared to the lowest [107]. Self-reported omega 3 supplementation has also been associated with a higher likelihood of natural conception [108]. Moreover, its supplementation may improve insulin resistance and the glycemic profile in women diagnosed with PCOS [109,110].

Wise et al. [100] conducted an observational study that included two cohorts with different food cultures: Danish women, who typically followed the Prudent diet (similar to the MD), and North American women, who usually followed a Western diet. On the one hand, the North American women’s ω3 intake enhanced their fertility, but it was unrelated to the Danish women’s ability to conceive, whose ω3 intake was usually high. On the other hand, higher TFA intake was linked with decreased fecundity for the North American women, while the Danish women’s TFA consumption, which was usually low, was unrelated to fecundity.

As with female fertility, it is well established that the impact of fats on male fertility depends on the fats’ quality. The higher total fat intake reported using FFQs has been negatively associated with the sperm concentration and total sperm count [111]. On the one hand, TFAs have been inversely correlated with seminal characteristics in observational studies that measured TFA levels in seminal plasma [112]. Similar results have been reported for saturated fatty acids (SFAs), because observational studies measuring with FFQs have found a lower sperm concentration and total sperm count with higher SFA intake [111,113]. A significant drop has been reported in the sperm concentration when more than 0.54% of the calories (2nd quartile) are derived from TFAs according to FFQs [114]. Similar effects have been observed in SFA consumption, and the total sperm count and concentration appear to lower when more than 10% energy is obtained from SFAs [113]. According to dietitians’ recommendations, this is the maximum percentage of energy that should be derived from SFAs [115]. Moreover, as more energy was obtained from this fatty acid type, a lower sperm concentration and total sperm count were observed, with an approximate 60% decrease in both parameters in cases with the highest SFA intake compared to those with the lowest intake [113]. It has been suggested that TFAs impair semen characteristics by decreasing the amount of PUFAs, mainly DHA, in sperm membranes because their high intake may interact with the enzymes involved in the fusion of PUFAs with the spermatozoa membrane [116,117,118]. The mechanisms by which SFAs may affect seminal properties remain unclear [113].

On the other hand, polyunsaturated fatty acids (PUFAs), including ω3, are key components of spermatozoa and play a role in the fluidity and flexibility of the membrane and also contribute to appropriate fertilization [116]. It has been reported that infertile men present lower blood and spermatozoa DHA and EPA levels than fertile men [119]. Positive effects of ω3 intake [107,111] and supplementation [120] have been reported in IVF patients. A randomized controlled trial, in which 238 men with idiopathic oligoasthenoteratospermia were randomized to either the placebo or the treatment group (DHA + EPA supplementation) for 32 weeks, reported a significant increase in the sperm concentration [120]. These results were confirmed by two systematic reviews, which concluded that supplementation and ω3 intake could enhance seminal parameters [121,122]. On another note, it has been suggested that improvements in semen quality may not be evident until 4 weeks after ω3 supplementation and may be dose dependent [123]. The positive effects of ω3 on male fertility may be due to the protective role of these fats against reactive oxidative species (ROS), considering that ROS may alter the spermatozoa membrane composition, including the ω3 concentration, and are the cause of infertility in 30–80% of men [121].

In addition, total TFAs and SFAs positively correlate with the odds of asthenozoospermia and a smaller testicular volume, while ω3 fatty acids lower these odds and are associated with a larger volume [124,125].

Only one study attempted to establish a relation between fatty acid intake by the male partner and pregnancy [126]. In that study, 697 couples who did not use reproductive assistance were followed up until pregnancy or for 1 year after enrolment. Surprisingly, no association between the fatty acid levels of the male partner and achieving natural pregnancy was reported.

As previously stated, incorporating moderate amounts of low-fat dairy products is an important aspect of the DASH diet. Research into the relation between low-fat dairy products and fertility was analyzed in an 8-year elegant long-term follow-up study with 18,555 women and their attempts to become pregnant. Infertility was reported by 438 women. This work observed how abundant consumption of low-fat dairy products increases the risk of developing anovulatory infertility [127]. In particular, consuming five to six servings of low-fat dairy products per week was reported to increase the anovulation risk by 86% compared to consuming one such serving or less per week (relative risk (RR) 1.86, 95%CI 1.19–2.91, *p* value 0.002). Due to the process of extracting fat, low-fat dairy products contain a higher IGF-I level, which has been linked with negative effects on ovulatory function [128]. It has also been speculated that an altered level of this protein may favor PCOS symptoms [129,130].

Unlike low-fat dairy products, high-fat dairy products might be considered to benefit ovulatory fertility and PCOS, possibly because of the smaller amount of IGF-I and, in addition, the larger amount of estrogens [127,128,131], which could exert a regulatory effect on IGF-I [132].

On the contrary, sperm parameters seem to improve with a high intake of low-fat dairy products [133,134]. A prospective observational study with a cohort of 155 subfertile men reported a 9.3% increase in progressive motility [135]. The authors suggested that enhanced sperm parameters may be due to the larger amount of IGF-I in such foods being associated with the molecular processes and protective effects involved in Leydig cells [136,137].

### 4.2. Protein

The effect of protein on fertility appears to be influenced by its source. Research findings suggest that increased dairy protein consumption is associated with a reduced antral follicle count in women seeking treatment at infertility clinics [138].

Other sources have been studied, such as the animal protein typically found in meat (which is a major component of the Western diet) and the vegetable protein that is commonly found in vegetarian and vegan diets (mainly in soy). An observational study included 18,555 women with no prior infertility issues who were followed up for 8 years while they attempted to become pregnant. Of these women, 438 experienced ovulatory infertility. In that study, the highest animal protein consumption, as reported using a baseline questionnaire, compared to the lowest was associated with a higher ovulatory infertility risk (RR 1.39, 95%CI 1.01–1.90, *p* = 0.03). In contrast, the highest vegetable protein consumption compared to the lowest was linked with a lower ovulatory infertility risk (RR 0.78, 95%CI 0.54–1.12, *p* = 0.07). Substituting 5% of the total energy intake for vegetable protein instead of animal protein has been linked with a reduction in the ovulatory infertility risk [139]. Vegetal protein consumption also seems to have an impact on reproductive hormones. In particular, the most recent prospective study observed that healthy participants with regular cycles in the lowest tertile of vegetal protein consumption had lower progesterone concentrations (−18.0%, 95%CI 30.2–3.6) and higher FSH levels (3.8%, 95%CI 0.2–7.6) compared to those in the second tertile [140].

There is limited information in the literature about male fertility. In 2010, a randomized crossover study was conducted to observe the effects of low-isoflavone ethanol-washed soy protein isolate (*n* = 31) and high-isoflavone soy protein isolate (*n* = 25) powder supplementation for 57 days on semen quality. The study results indicated that there is no significant effect [141]. Nevertheless, a recent cross-sectional study, in which the dietary data were reported using FFQs, found that similar to female fertility, a higher intake of plant-based protein lowers the oligozoospermia risk in subfertile men (OR 0.31, 95%CI 0.14–0.65, *p* = 0.002) compared to the lowest vegetable protein intake. On the contrary, high animal protein intake increased the oligozoospermia risk (OR 2.42, 95%CI 1.13–5.19, *p* = 0.002) and the teratozoospermia risk (OR 3.97, 95%CI 0.97–16.16, *p* = 0.05) [142].

### 4.3. Carbohydrates

According to the literature, studies that have focused on the total amount of carbohydrate and glycemic load have observed that higher levels of these nutrients negatively impact fertility [143,144,145]. In a large cohort of 18,555 women, Chavarro et al. [143] observed a 1.91-fold (95%CI 1.27–3.02) higher ovulatory infertility risk in women who consumed more carbohydrates. It has also been reported that high glucose levels may prolong the time to achieve naturally conceived pregnancy [145]. Diets with small amounts of carbohydrates appear to especially benefit woman with PCOS. A systematic review [146] found that low-carbohydrate diets, including ketogenic diets, have a positive effect on this population’s ovarian regulation.

The potential adverse association between excessive carbohydrate consumption and infertility may be attributed not only to carbohydrates but also to their impact on insulin levels, which can potentially result in insulin disorders, such as hyperinsulinemia and insulin resistance [147]. Both these insulin disturbances correlate positively with hyperandrogenism in PCOS women. Larger amounts of androgens could lead to an alteration in gonadotropin production and oocyte maturation, which may result in anovulation and, thus, fertility impairment [148,149].

Studies on female fertility have reported that ovarian function does not seem to be disturbed by sweet beverage intake [150]. However, it has been reported in observational studies using FFQs that obese and overweight women with PCOS consume larger amounts of sweet foods and sugary drinks than women without this condition [151]. For men, the literature suggests that simple sugars may negatively affect semen quality [84,152]. According to the results of Eslamian et al. [90], men who consumed larger quantities of sweet food were at twice (OR 2.05, 95%CI 1.09–2.26, *p* = 0.046) the asthenozoospermia risk. However, to the best of our knowledge, the only publication that assessed the relation between pregnancy and men’s sweet food intake found no association between the two. Nevertheless, it is worth mentioning that no significant association between sweet food consumption and semen parameters was observed in that study [153].

## 5. Micronutrients

### 5.1. Folic Acid and Vitamin B12

The B vitamins present in fruits and vegetables, such as folic acid (B9), appear to have a protective effect against ovulatory infertility. Chavarro et al. [154] reported that participants who self-reported the highest folic acid intake using FFQs presented a lower ovulatory infertility risk compared to those with the lowest consumption. In fact, IVF women who consumed 400 μg of supplemental folate per day, as reported using FFQs, presented a modestly lower follicle count (approx. 1.5 fewer follicles) compared to those who consumed 800 μg of supplemental folate per day [155]. An observational study also observed a 20% lower live birth rate in women who took less than 400 μg of folate acid supplementation per day compared to those who consumed more than 800 μg per day [156]. Moreover, folic acid is recommended by the authorities for pregnant women or for women planning pregnancy because it is essential for proper development of fetuses’ neural tubes [157].

Serum folic acid levels have been observed to be higher in men with normal semen quality than in those with altered semen characteristics. Indeed, an increase of one unit of folic acid in men has been associated with a 17% reduction in the prevalence of altered semen quality [158]. Daily folic acid supplementation for men with oligozoospermia and the MTHFR 677T genotype results in improved semen quality and in higher spontaneous conception and live birth rates compared to placebo supplementation [159]. Nevertheless, most research into the effects of folic acid on semen parameters has been conducted with the addition of zinc. In the most recent meta-analysis, the authors concluded that supplementation of zinc plus folic acid has a positive effect on subfertile men’s sperm quality [160].

The data from the literature suggest that vitamin B12 is positively linked with female fertility [161]. According to the literature, over half of the women who attend a fertility clinic present deficient serum vitamin B12 levels [162]. Vegetarians and vegans are at higher risk of vitamin B12 deficiency because small amounts of this vitamin are found in plants. In subfertile patients, higher B12 levels in blood have shown better embryo quality [163]. Furthermore, a retrospective study with 100 women (154 ART cycles) reported that women with the highest serum vitamin B12 levels have better chances of live births (RR 2.04, 95%CI 1.14–3.62, *p* = 0.0008) than those with the lowest levels [164]. Higher B12 intake has also been associated with higher live birth rates [156]. Moreover, low vitamin B12 serum levels have been linked with recurrent pregnancy loss [165,166], perhaps because this vitamin, together with folate, is necessary to convert homocysteine into methionine, and high homocysteine levels may result in miscarriage [167,168]. A recent meta-analysis has also suggested that vitamin B12 serum levels in pregnant women may play a role in the offspring’s epigenetics. However, this has not yet been linked with children’s health outcomes in any way [169].

Several authors consider adequate vitamin B12 intake to be crucial for preventing male infertility [170], and they have proposed this molecule supplementation as an approach to improve semen quality in infertile men [171,172]. In general, supplementation with vitamin B12 and its derived compounds appears to increase the sperm count in subfertile men [173]. Regarding vitamin B12 levels, specifically in studies that have compared individuals on a plant-based diet with omnivores, vegetarians present lower serum and seminal cobalamin levels, which do not appear to be related to seminal quality. Nevertheless, in both groups (vegetarian and nonvegetarian), azoospermic men present lower vitamin B12 levels in seminal plasma than normo- and oligozoospermic men [174,175]. In addition, appropriate B12 intake appears to be particularly important for semen characteristics when men present specific genetic variations, such as MTHF C677T polymorphism [176]. Several mechanisms related to how vitamin B12 enhances semen parameters are suggested in the literature, such as antioxidant and anti-inflammatory action or a drop in homocysteine, which is associated with reproductive impairment [173].

### 5.2. Vitamin D

The primary sources of vitamin D for humans are mainly certain types of fish, mushrooms, and fish liver oils, which are commonly consumed in the MD. Cheese, beef liver, and dark chocolate are other sources with more modest amounts of vitamin D [177].

The literature on female fertility suggests that vitamin D participates in both ovarian and endometrial function by taking part in follicular development and embryo implantation processes [178,179]. Nevertheless, the correlation between vitamin D and improved reproductive parameters is still ambiguous [180,181,182,183,184]. Findings are contradictory, and studies are heterogeneous because they do not present the same threshold of vitamin D levels and have been performed in different countries. Moreover, optimal vitamin D levels may change depending on the target population [185]. The literature suggests that once the minimum vitamin D intake requirement is met, it has no advantageous impact on female fertility [186,187]. Interestingly, vitamin D deficiency has been associated with insulin resistance and women with PCOS, and both conditions are related to infertility [188]. Several clinical trials have evaluated the impact of vitamin D supplementation on PCOS women and observed an alteration in sex hormone ratios. This indicates that vitamin D might ameliorate hormonal alteration in PCOS and result in decreased insulin resistance, which might favor fertility in the women with this condition [189,190].

For male fertility, although no clear consensus in the literature exists, mostly due to data heterogeneity, the general agreement is that vitamin D enhances semen parameters, especially motility in asthenozoospermic infertile men [191,192,193,194]. A randomized, triple-masking clinical trial with 330 infertile men, in which the treatment group received 1400 IUs of vitamin D daily for 150 days, reported a 20% higher live birth rate in oligozoospermic men compared to the placebo group [195].

### 5.3. Phytoestrogens

Phytoestrogens are found mainly in soy. Vegetarians consume a large quantity of this legume due to its high protein content and versatility [196]. Some concern has been voiced about the effect of soy on male and female reproductive systems, and soy was initially considered a reproductive toxicant [187]. However, different studies have demonstrated a positive correlation between soy consumption and ovulation [197] and live birth rates [198].

Phytoestrogens, including isoflavones, have been reported to exert a positive effect on clinical outcomes. A prospective cohort study with 315 subfertile women observed in multivariable-adjusted models that the highest intake of both soy and isoflavone leads to a significantly higher clinical pregnancy rate and live birth rate compared to the lowest intake in observational studies using FFQs [198]. In addition, their inclusion in strategies such as progesterone supplementation in the luteal phase [199] and treatment with citrate for ovarian stimulation [200] has been suggested to enhance reproductive outcomes. In natural conception, high urinary isoflavone levels do not appear to be associated with increased fecundity [201], and even a slight reduction in the number of women achieving pregnancy has been reported with high isoflavone intake, as reported with FFQs [202].

For male fertility, the data in human studies are controversial [203]. Whereas in healthy men, semen characteristics remained unchanged after supplementation with 40 mg of isoflavones for 2 months [204], in subfertile men, a negative effect of soy food and isoflavone consumption on the sperm concentration was reported [205]. Nevertheless, this diminished semen quality may not be extrapolated to reproductive success because soy intake, as reported in a questionnaire, by the men in subfertile couples has not been reported to be related to any fertility outcomes [206].

### 5.4. Antioxidants

Antioxidants are molecules that delay oxidation. They are primarily found in plants, which include fruits, nuts, and vegetables [207]. A balance between antioxidants and ROS is required for proper reproductive function. ROS in proper amounts are involved in several female fertility aspects, such as ovarian hormonal function and oocyte maturation [208], and also in male fertility aspects, such as in the final steps of spermatogenesis, hyperactivation, and acrosome reaction [209,210]. Nevertheless, an imbalance between these molecules and antioxidants leads to oxidative stress, which has been associated with ovarian aging [211], PCOS [212], endometriosis [213], miscarriage [214], sperm DNA damage, and apoptosis [210].

In the most recent Cochrane reviews [215,216], the authors claim that there is little evidence to suggest that antioxidants improve male and female fertility. The most recent meta-analysis [215] searched for an association between female reproductive outcomes and oral supplementation with antioxidants and included 35 trials. It found low-quality evidence to suggest improved clinical pregnancy, from 19% in women with no treatment or administered a placebo to 25–30% in women taking oral antioxidants. The data suggest a 5–17% increase in the live birth rate for subfertile women using antioxidants compared to the placebo or nontreatment group. Regarding male fertility, the most recent meta-analysis [216] indicates low-certainty evidence of a higher clinical pregnancy rate in men on antioxidant supplements (OR 1.89, 95%CI 1.45–2.47, *p* < 0.001), with no evidence for adverse events.

In men, a meta-analysis and a systematic review with strict inclusion criteria of well-designed, double-blind, and placebo-controlled trials concluded that coenzyme Q10, selenium, and combinations of others like L-carnitine + acetyl-L-carnitine or folic acid + zinc show improvements in seminal parameters [217]. In line with these results, recently, another meta-analysis and a systematic review also detected that supplementation with these combinations of antioxidants (L-carnitine + acetyl-L-carnitine and folic acid + zinc) is beneficial in subfertile men [218].

### 5.5. Gluten

Celiac disease has been associated with a higher incidence of fertility issues. It has been reported that women with celiac disease are more likely to experience amenorrhea, premature menopause, recurrent pregnancy loss, spontaneous miscarriage, endometriosis, and PCOS [219,220]. Research has shown that women experiencing unexplained infertility are three times more likely to have celiac disease than fertile women. However, it is important to note that the sample size used in that study was relatively small. Thus, further research is needed before implementing a celiac disease test in routine reproductive clinic practice [221]. Although eliminating gluten from the diet improves reproductive outcomes in women with nonceliac gluten sensitivity and celiac women [220,222], it is not recommended for the general population because it may cause malnutrition, and there is no evidence of any benefit [223,224].

Data available on the correlation between gluten intake and male fertility are limited. Celiac disease has been associated with reduced libido and sexual activity in men [225]. It would also seem that sexual hormone levels are affected by this condition but return to normal when individuals follow a gluten-free diet [226]. However, more research is needed to fully address this topic.

## 6. Limitations and Future Directions

Certain difficulties in drawing conclusions about nutrition and fertility should be considered. First, healthier diets are usually adopted by people with healthy lifestyles, including practicing considerable physical activity, which could also influence fertility. Moreover, infertility may lead to a change in several lifestyle behaviors that are not usually contemplated [88]. On another point, most studies are observational and based on questionnaires, which may entail unreported data and may limit the causal inference process [98]. In addition, there is considerable heterogeneity in the scientific literature, with studies conducted in several countries with their own traditional gastronomy, studies of different durations, and studies using distinct types of questionnaires that may vary in the amount of food reported and the duration of reported consumption (e.g., the past 6 months or in the past year). One of the challenges in studying the impact of diet on fertility is the lack of a defined minimum period of exposure required to measure potential changes in the parameters under analysis. In the case of men, it can be estimated that any intervention should last a minimum of 2 to 3 months to identify changes in semen analysis, given that the spermatogenesis cycle lasts approximately 2.5 months [227,228]. However, in women, this cycle of generation of new germ cells does not exist [229]. Thus, if the diet has an impact, it is on other parameters that are related to the possibility of pregnancy, such as weight [230] and the ability to ovulate spontaneously [57,99,127], among others. Furthermore, there are different measurements (FFQs and serum levels) for specific micronutrients, such as ω3, folic acid, and vitamin B12. Omega 3 intake has been observed to have a linear association with serum ω3 levels [231]. For folic acid, a meta-analysis that extracted data from 108 studies estimated that for every 100 µg/day intake of this vitamin, the serum folate concentration increases by 11.6% [232]. Finally, a study that monitored serum folate and B12 levels concluded that they correlate modestly with the intake of these vitamins [164]. Many studies that have assessed the relation between diet and male infertility relied on a semen analysis, which may not be strictly linked with an individual’s ability to conceive [114]. More appropriate and robust studies that address these limitations are needed to provide more reliable evidence.

## 7. Conclusions

Trend diets seem to impact women’s and men’s fertility. Although there is still limited and ambiguous information about certain nutrients, a balanced, healthy diet (rich in ω3, plant protein, vitamins, and antioxidants, with limited TFAs) is generally recommended to achieve pregnancy. It is worth noting that women who plan to become pregnant are strongly recommended to begin taking folic acid supplements. Further research with stronger evidence is needed to provide general and individualized dietary advice for women and men who wish to attempt to conceive.
